# Interaction between Antibacterial Peptide Apep10 and *Escherichia coli* Membrane Lipids Evaluated Using Liposome as Pseudo-Stationary Phase

**DOI:** 10.1371/journal.pone.0164594

**Published:** 2017-01-04

**Authors:** Wenting Tang, Chuanfen Pu, Man Li

**Affiliations:** School of Food Science and Engineering, Qingdao Agricultural University, Qingdao, 266109, China; Griffith University, AUSTRALIA

## Abstract

Liposomes constructed from *Escherichia coli* membrane lipids were used as a pseudo-stationary phase in capillary electrophoresis and immobilised liposome chromatography to evaluate the interaction between antibacterial peptide (ABP) Apep10 and bacterial membrane lipids. The peptide mobility decreased as the concentration of liposomes increased, providing evidence for the existence of this interaction. The binding constant between Apep10 and the *Escherichia coli* membranes lipid liposome was higher than that of Apep10 with a mixed phospholipids liposome at the same temperature. The capillary electrophoresis results indicate that the binding ability of Apep10 with a liposome was dependent on the liposome’s lipid compositions. Thermodynamic analysis by immobilised liposome chromatography indicated that hydrophobic and electrostatic effects contributed to the partitioning of Apep10 in the membrane lipids. The liposomes constructed from bacterial membrane lipid were more suitable as the model membranes used to study dynamic ABP/membrane interactions than those constructed from specific ratios of particular phospholipids, with its more biomimetic phospholipid composition and contents. This study provides an appropriate model for the evaluation of ABP-membrane interactions.

## Introduction

Antibacterial peptides (ABPs) have attracted much attention due to their potential to overcome bacterial resistance and are promising candidates for novel antibiotics [1], [2]. It is well established that the first stage of ABPs’ action is to combine with the bacterial cell membrane [3–5]. Therefore, knowledge of this interaction is vital to understand their antibacterial mechanism. However, the natural cell membrane has a very short lifespan, which makes it an imperfect candidate for research. To overcome this shortcoming, self-assembled vesicles known as liposomes have been widely used as model membranes, due to the structural similarities between liposomes and the natural cell membrane, both of which are phospholipid bilayers [6–9].

Immobilised liposome chromatography (ILC), a technique in which liposomes are immobilised in stationary phases for liquid chromatography, has been studied as a membrane analysis technique [10–13]. However, the liposomes used for this method are usually constructed from specific ratios of particular phospholipids that cannot mimic the fine balance of phospholipid compositions and contents in real cell membranes. The highly regulated lipid composition of natural bacterial cell membranes is a vital component in the evaluation of ABP-membrane interactions. Thus, the diversity of bacterial membranes requires more appropriate stationary phases that can take account of their specific lipid compositions.

Capillary electrophoresis (CE) has been used as an effective tool to investigate the specific or non-specific interactions between bioactive components and their targets [14–15]. Liposomes have been used as a pseudo-stationary phase in CE for the analysis of analyte-membrane interactions using peak shift methods. By supplementing the background electrolyte with a series of concentrations of liposomes, variations in the mobility of the bioactive component can be measured [16]. This method has the advantages of high analysis efficiency, low sample volume and low reagent consumption. The liposomes can disperse freely in the running buffer, which involves few steric restrictions and better mimics the interaction between bioactive components and lipid vesicles [17].

In a previous study, we developed an effective method of *Escherichia coli* cell membrane chromatography for the separation of ABPs, based on the affinity interaction between ABPs and bacterial cell membrane liposomes [18]. Detailed information regarding the interaction between endogenous bacterial membrane lipids and ABPs may be useful for screening and designing ABPs. The aim of this study was to investigate the interaction between *E*. *coli* membrane lipid liposomes and the antibacterial peptide Apep10 using the liposome as a pseudo-stationary phase in CE and ILC. The binding constant and thermodynamic parameters of Apep10 interacting with the *E*. *coli* membrane lipid liposome were also compared with those of the peptide with the specific mixed phospholipid liposome to analyse the interaction difference.

## Materials and Methods

### Chemicals

Egg-yolk phosphatidylcholine (EYPC) and 1, 2-Dimyristoyl-sn-glycero-phosphatidylglycerol (DMPG) were purchased from A. V. T. Pharmaceutical (Shanghai, China). The antibacterial peptide Apep10 (GLARCLAGTL), screened from boiled-dried anchovies by immobilized bacterial membrane liposome chromatography, was provided by the School of Food Science and Engineering, Qingdao Agricultural University. *Escherichia coli* ATCC 25922 was obtained from the China Center of Industrial Culture Collection (CICC, Beijing, China). Luria-Bertani (LB) broth and agar medium were purchased from Baisi Biotechnology Co., Ltd. (Hangzhou, China). All other chemicals were of analytical grade and used without further purification.

### Extraction of *E*. *coli* membrane lipid

*E*. *coli* was cultured aerobically in LB broth at 37°C, which is the optimal growth temperature for the strain. The species was transferred weekly on agar media to keep the microorganisms viable and maintained at 4°C. Total lipid was extracted from membranes of *E*. *coli* according to the previously reported method with some modifications [19]. *E*. *coli* ATCC 25922 cultured in 3 L of LB broth at 37°C for 24 h was harvested by centrifugation (3,000 *g*, 5 min). The collected cell pellets were washed with Tris buffer (25 mM, pH 7.5) and resuspended in 50 ml of the same buffer, and 150 ml of a 2:1 (v/v) methanol-chloroform mixture was added to the cell suspension. The whole was sonicated for 60 s. A further 50 ml of distilled water and 50 ml of chloroform were then added. The cell/solvent sample was again sonicated for 60 s and allowed to stand for 1 h. The lipid extract was separated with a separatory funnel, dried by rotary evaporation under vacuum, and stored at -18°C.

### Preparation of *E*. *coli* membrane lipid liposomes

Liposomes were prepared with the rotary evaporation method, as described previously [20]. Briefly, the *E*. *coli* lipid extract or the mixture of EYPC/DMPG (2:1, w/w) was dissolved in a chloroform/methanol mixture (1:1, v/v) in a round bottom flask. The solvent was dried in a rotary evaporator under reduced pressure, and the remaining solvent was removed under high vacuum overnight for 2 h. The dried film was hydrated with phosphate buffer (10 mM, pH 7.0) to a final total lipid concentration of 8 mg/ml. The suspension was sonicated in an ice bath with a sonifier cell disruptor (Xinchen, Nanjing, China). Unilamellar liposomes were obtained by extruding the sample through a polycarbonate membrane with 100 nm pores (Millipore, Bedford, MA) using a manual extruding device (Morgec, Shanghai, China). The liposomes were freshly prepared and serially diluted before CE experiments.

### CE analysis

The CE analysis was performed on a Beckman P/ACE MDQ (Beckman Coulter, Pasadena, CA) coupled with a 65 cm (effective length of 57 cm to the detector) × 75 μm I.D. uncoated fused-silica capillary (Hengyao Chromatogram Equipment Co., Ltd., Shanghai, China). The new capillary was first conditioned with 1 M NaOH for 30 min and redistilled water for 10 min. Before each injection, the capillary was consecutively washed with redistilled water for 1 min, 1 M HCl for 1 min, redistilled water for 1 min, 1 M NaOH for 1 min, redistilled water for 1 min, and the running buffer for 5 min at a pressure of 20 psi pressure. The peptide (1 mg/ml) was dissolved in the phosphate buffer. The buffer solution (phosphate buffer, 10 mM, pH 7.0) was freshly prepared daily by redistilled water. The running electrolyte and the injected peptide solution were filtered through a 0.22 μm membrane filter, and then degassed before use. The sample (1 mg/ml) was injected by positive pressure at 0.8 psi for 6 s. The separation voltage was 25 KV. The detector wavelength was set at 196 nm. The mobility shift assay was conducted using a phosphate buffer (10 mM, pH 7.0) containing a series of concentrations of liposomes as the running buffer. The measurement was repeated three times. Acetone was used as the neutral electroosmotic flow. The Apep10 mobilities were corrected for viscosity effects caused by liposomes in the phosphate buffer according to the following equation [21]:

*μ* = *μ*_0_*η* / *η*_0_ where *η* and *η*_*0*_ are the viscosities of background electrolyte in the presence and absence of liposomes, respectively. *μ*_*0*_ and *μ* are the determined and corrected electrophoretic mobility, respectively. Viscosity was measured with an automated Ubbelohde-type capillary viscometer (Schott Gerate, Hofheim, Germany), controlled by a thermostatted water bath. The viscosity was measured at the temperature that corresponded to the CE experiments.

The effective electrophoretic mobility of the peptide was calculated according to the following equation:

$$\mu_{eff}=\mu_{ap}-\mu_{ac}=\frac{L_{d}L_{t}}{V}(\frac{1}{t}-\frac{1}{t_{ac}})$$

Where *μ*_*eff*_ is the effective mobility of the peptide, *μ*_*ap*_ is the apparent mobility of the peptide, *μ*_*ac*_ is the mobility of acetone, L_d_ is the length of the capillary from injection to detection, L_t_ is the total length of the capillary, V is the separation voltage, t is the migration time of the peptide and t_ac_ is the migration time of acetone.

The Scatchard analysis was performed, and the binding constant (K) was estimated by plotting Δ*μ*/[C] versus Δ*μ* according to the following equation:

$$\Delta \mu =\mu_{effi}-\mu_{eff\;0}$$

$$\frac{\Delta \mu }{C}=-K\Delta \mu +K\Delta \mu_{\max}$$

Where Δ*μ* is the effective mobility difference, *μ*_*effi*_ is the effective mobility of the peptide in the running buffer with various concentrations of the liposome, *μ*_*eff0*_ is the effective mobility of the peptide in the running buffer without liposome, Δ*μ*_max_ is the maximum value of the effective mobility difference, C is the concentration of the liposome in the running buffer and *K* is the binding constant [22].

### Determination of thermodynamic analysis of Apep10 binding to bacterial membrane lipids by ILC

Determination of thermodynamic analysis of Apep10 binding to bacterial membrane lipids was performed by immobilized liposome chromatography (ILC) under different temperatures. Preparation of the immobilised liposome stationary phase was conducted according to a previously reported method [18]. The obtained stationary phase was packed into a stainless steel column (150 mm length × 4.6 mm i.d.) with the slurry packing method. Sodium phosphate buffer (PBS, pH 7.2, 10 mM) containing 50 mM NaCl was used as the mobile phase. The column was equilibrated with the buffer before sample injection. The flow-rate was 0.5 mL/min and the UV monitor wavelength was 215 nm. Enthalpy change (⊿H), entropy change (⊿S), and free energy change (⊿G) were evaluated the following equations:

*Ink* = −Δ*H* / *RT* + Δ*S* / *R* Δ*G* = Δ*H* − *T*Δ*S k* = (*V*_*R*_ − *V*_0_) / *A* Where *k* is the capacity factor; R is the gas constant; T is the absolute temperature; V_R_ is the retention volume of the peptide; V_0_ is the void volume of the reference molecule (NaNO_3_), which does not interact with the liposomes or the silica; and A is the amount of immobilised phospholipid amount in the column, which was determined by phosphorus analysis [23].

## Results and Discussion

### CE analysis

The initial interaction of ABPs with negatively charged model lipid membranes has been reported to be one of the most important attributes of ABPs when they execute their antibacterial activities. The interactions of ABPs with liposome lipid bilayers have been extensively investigated to elucidate the mechanisms of action of ABPs. Some ABPs may penetrate the lipid bilayer, and others may bind to the head group region parallel to the plane of the bilayer; they may further perturb the hydrophobic region and induce a rapid flip-flopping of the phospholipids. The different phospholipid compositions of the bilayers of the liposomes may contribute to these differences in interaction. Generally, positively charged ABPs interact more strongly with negatively charged membranes than with neutral membranes [7, 8, 24, 25]. However, there have been few reported comparisons between endogenous bacterial lipids and negatively charged phospholipids, which were most commonly used to mimic the cell membrane.

**Fig 1:**
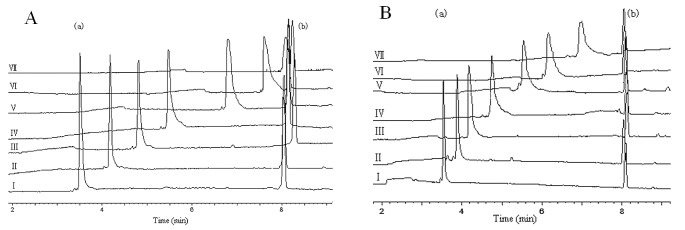
Electropherograms of Apep10 and acetone in the running buffer with various concentrations of (A) the *E*.*coli* membrane lipid liposome and (B) the mixed phospholipids liposome at 303 K. The liposome concentrations (mg/ml) were: Ⅰ, 0; Ⅱ, 0.1; Ⅲ, 0.2; Ⅳ, 0.4; Ⅴ, 0.8; Ⅵ, 1.2; Ⅶ, 1.6. The electrophoresis conditions used were as follows: injection, 0.8 psi for 6 s; separation voltage, 25 kV; detection, UV detection at 196 nm; and capillary, 65 cm × 75 μm inner diameter. Peaks (a) and (b) were Apep10 and acetone, respectively.

The viscosity of the buffer solution in the presence of the liposomes affects the mobility of the analytes. The increased viscosity decreases the mobility of the charged analytes. As a result, the mobility of Apep10 was corrected according to Franzen et al [26]. The electropherograms of Apep10 and acetone in the running buffer with various concentrations of the *E*. *coli* membrane lipid liposome and the mixed phospholipid liposome at 303 K are shown in Fig 1. Under both circumstances, the migration time of acetone, which was used as the neutral electroosmotic flow, caused almost no change. The fact that the retention characteristics of acetone were not affected by increasing the concentration of the two liposomes indicates that the interaction between acetone and the liposomes was weak. It should also be noted that at pH 7.0, the surface of the capillary wall was negatively charged due to ionisation of the silanol on the wall surface [27]. The two kinds of liposomes in the buffer were also negatively charged (the surface potentials of the *E*. *coli* membrane lipid and the mixed phospholipids liposomes were −5.7±0.8 mV and −13.8 mV, respectively). There was a balance between the interaction of the liposomes and the capillary wall, including electrostatic repulsion and attraction of the liposomes onto the wall due to hydrophobic and coulombic interaction [28]. No significant change in the neutral electroosmotic flow mobility was observed in the presence of different concentration of the liposomes (Fig 1), indicating that the interaction between the liposomes and the capillary wall did not affect the determination of mobility. The peak of Apep10 appeared before acetone in the pH 7.0 running buffer, suggesting that the peptide was positively charged. This was consistent with our previous findings, which showed that the total net charge of Apep10 was +1 in a neutral environment [18]. The charge state of Apep10 facilitates its binding with the anionic model liposomes or bacteria cell membranes.

In the presence of both types of liposomes, the migration time of Apep10 increased with increasing concentrations of liposomes. In addition, the peaks of Apep10 became lower and wider at higher concentrations of the liposomes, which might have been caused by the stronger interaction between Apep10 and liposomes. The interaction between positively charged Apep10 and the capillary wall might also have contributed to the wider peaks. According to Corradini et al [29], the presence of liposomes could reduce to a certain extent the undesirable interactions between basic protein and the capillary wall. Apep10 is positively charged as a basic protein. Here, the liposomes might have had the same effect on the interactions between the peptide and the capillary wall.

**Fig 2:**
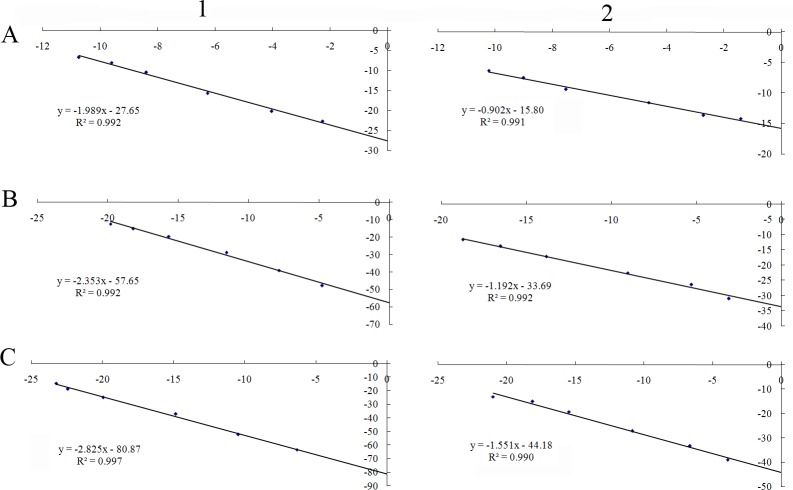
The Scatchard plot of Apep10 in the running buffer containing a series of concentrations of (1) the *E*. *coli* membrane lipid liposome and (2) the mixed phospholipids liposome. A, 283 K; B, 293 K; C, 303K.

The experimental and calculated data of the electrophoresis experiment are shown in Table 1. The reported *K* values are averages from three replicate experiments. The binding constants *K* at 303 K, obtained by plotting Δ*μ*/[C] versus Δ*μ*, were 2.8252 ml/mg for the *E*. *coli* membrane lipid liposome and 1.551 ml/mg for the mixed phospholipids liposome (Fig 2). The approach is valid only when the binding stoichiometries of the peptide to the liposome is equal to 1:1. The accessible surface area of the liposome might be larger than that of the peptide. Therefore, the liposome can bind to a number of Apep10 molecules. Consequently, the binding constant measured here was a total value, which referred to all the binding capacities of the sum of the binding sites [30]. The bonding constants for both systems increased as the temperature increased, indicating enhancement of the capacity of Apep10 binding to the liposomes. It also suggested the involvement of non-ionic interactions in the binding of Apep10 to the liposomes [31]. The bonding constant of Apep10 with *E*. *coli* membrane lipids was higher than that with mixed phospholipid liposomes at the same temperature. The comparison of data demonstrated that Apep10 interacted more strongly with the *E*. *coli* membrane lipid liposomes than with the mixed phospholipids liposomes. It is believed that the composition of the target membrane has a significant effect on the extent of binding and efficacy of action of ABPs. The lipids from *E*. *coli* membranes were reported to contain about 85% of phosphatidylethanolamine (PE) [19], while the mixed phospholipids we used to construct liposome comprise one third of DMPG and two thirds of EYPC. The variation in the binding ability of Apep10 to both liposomes, revealed by comparison of the binding constants, was dependent on the differing lipid compositions of the target membranes. Liposome constructed from endogenous bacterial membrane lipids might be more suitable as model membranes for evaluating the binding interaction between ABPs and the membrane bilayers involved in the antibacterial action.

**Table 1:** Experimental and calculated data of the CE analysis for Apep 10 at 303K.

Liposome type	Liposome concentration (C, mg/ml)	Migration time (t, min)		Apparent mobility (*μ*_*ap*_, 10^−3^·cm^2^·V^-1^·min^-1^)		Effective mobility (*μ*_*eff*_, 10^−3^·cm^2^·V^-1^·min^-1^)	Effective mobility difference (Δ*μ*, 10^−3^·cm^2^·V^-1^·min^-1^)	Δ*μ*/C (10^−3^·cm^2^·ml·V^-1^·min^-1^·mg)
		acetone	Apep10	acetone	Apep10			
*E*. *coli* membrane lipid liposome	0	8.032±0.002^a^	3.525±0.003^a^	18.452±0.004^k^	42.039±0.030^m^	23.587±0.028^m^		
	0.1	8.050±0.002^b^	4.156±0.003^c^	18.409±0.003^j^	35.659±0.023^k^	17.250±0.021^k^	-6.337±0.047^k^	-63.365±0.466^a^
	0.2	8.246±0.002^k^	4.766±0.004^f^	17.973±0.003^a^	31.097±0.023^h^	13.124±0.024^i^	-10.462±0.050^i^	-52.311±0.251^b^
	0.4	8.110±0.002^f^	5.486±0.003^g^	18.274±0.003^f^	27.014±0.013^g^	8.740±0.010^g^	-14.847±0.019^g^	-37.117±0.048^d^
	0.8	8.148±0.002^i^	6.791±0.002^j^	18.189±0.005^c^	21.824±0.007^d^	3.635±0.002^d^	-19.952±0.030^d^	-24.940±0.037^g^
	1.2	8.129±0.001^h^	7.646±0.002^l^	18.231±0.002^d^	19.384±0.004^b^	1.153±0.006^b^	-22.434±0.034^b^-23.239±0.025^a^	-18.695±0.029^i^-14.524±0.015^k^
	1.6	8.226±0.002^j^	8.070±0.003^m^	18.017±0.003^b^	18.364±0.007^a^	0.348±0.004^a^		
Mixed phospholipid liposome	0	8.089±0.003^d^	3.526±0.003^a^	18.321±0.006^h^	42.031±0.032^m^	23.709±0.029^n^		
	0.1	8.033±0.003^a^	3.875±0.002^b^	18.449±0.006^k^	38.245±0.017^l^	19.796±0.018^l^	-3.913±0.015^l^	-39.132±0.150^c^
	0.2	8.065±0.002^c^	4.184±0.003^d^	18.375±0.005^i^	35.418±0.026^g^	17.043±0.023^j^	-6.667±0.058^j^	-33.333±0.289^e^
	0.4	8.098±0.001^e^	4.758±0.001^e^	18.302±0.003^g^	31.148±0.007^i^	12.846±0.004^h^	-10.864±0.033^h^	-27.159±0.082^f^
	0.8	8.128±0.002^h^	5.595±0.002^h^	18.234±0.005^d^	26.490±0.007^f^	8.255±0.003^f^	-15.454±0.033^f^	-19.318±0.041^h^
	1.2	8.114±0.002^fg^	6.216±0.005^i^	18.265±0.005^ef^	23.841±0.020^e^	5.576±0.020^e^	-18.133±0.013^e^	-15.111±0.010^j^
	1.6	8.117±0.006^g^	7.068±0.001^k^	18.259±0.014^e^	20.968±0.003^c^	2.709±0.011^c^	-21.001±0.037^c^	-13.125±0.023^l^

Results are expressed as mean value ± standard deviation (n = 3). Different letters (a, b, c, d…) in the same row represent a significant difference (*P*<0.05).

### ILC analysis

ILC analysis was performed to further illustrate the thermodynamic parameters of the interaction between Apep10 and bacterial cell membrane lipid. ILC has been reported as a promising method to investigate the partitioning of active substances into biomembranes [32], [33]. The thermodynamic parameters were analysed on the basis of capacity factor *k*, an index to assess the interaction between the liposome stationary phase and peptide (Table 2). The influence of temperature indicated the contribution of enthalpy to the interaction energy. The bilayers became less ordered as the temperature increased, eventually leading to the changes in enthalpy and entropy. The value of ⊿G was negative, indicating that the partitioning process was spontaneous. The partitioning of Apep10 in the *E*. *coli* membrane lipid liposome stationary phase exhibited higher *k* values than that in mixed phospholipid liposome under the same temperature, suggesting that it exhibited more interaction with the former, which was in accordance with the results of CE. The difference in the partitioning of Apep10 in both stationary phases may have arisen from the different compositions of the liposomes. According to Ross and Subramanian, the thermodynamic parameters determined were the sum of all of the thermal effects in the partitioning process. The values of ⊿H and ⊿S also indicated that the hydrophobic effect and electrostatic force contributed to the partitioning of Apep10 in the stationary phases [34].

**Table 2:** The thermodynamic parameters of Apep10 interacting with *E*. *coli* membrane lipid liposome and mixed phospholipid liposome.

Liposome	Temperature (K)	*k*(M^-1^)	*r*^2^	⊿H(KJ/mol)	⊿S(J/mol·K)	⊿G(KJ/mol)
*E*. *coli* membrane lipid liposome	0.00254	16.98±0.0047f	0.97±0.0012^a^	1.55±0.017^a^	27.48±0.043^a^	-9.26±0.0006^g^
	0.00251	17.04±0.0020^g^				-9.39±0.0004^e^
	0.00248	17.20±0.0006^h^				-9.53±0.0002^c^
	0.00245	17.30±0.0007^i^				-9.67±0.0001^b^
	0.00242	17.34±0.0002^j^				-9.81±0.0002^a^
mixed phospholipid liposome	0.00254	15.03±0.0013^a^	0.95±0.0047^a^	2.92±0.025^b^	29.97±0.066^b^	-8.87±0.0002^j^
	0.00251	15.26±0.0001^b^				-9.02±0.0003^i^
	0.00248	15.46±0.0058^c^				-9.17±0.0006^h^
	0.00245	15.64±0.0006d				-9.32±0.0009^f^
	0.00242	15.67±0.0087e				-9.47±0.0012^d^

Results are expressed as mean value ± standard deviation (n = 3). Different letters (a, b, c, d, e) in the same row represent a significant difference (*P*<0.05).

## Conclusion

In this study, CE and ILC analyses using liposomes as pseudo-stationary phases were established to explore the binding of antibacterial peptide Apep10 with an *E*. *coli* endogenous membrane lipid liposome and a mixed phospholipid liposome. The binding constants of Apep10 with the liposomes prepared from lipids derived from *E*. *coli* membrane lipids and mixed phospholipids were calculated and compared. A spontaneous partitioning process containing hydrophobic effect and electrostatic force might contribute to the action of Apep10. Our data suggest that the pseudo-stationary phase constructed from liposome could provide valuable information that would aid in the characterization of the interactions between ABPs and bacterial membrane lipids. Furthermore, the binding constants and capacity factor estimated support the conclusion that the composition of the target membrane is important for the binding action of ABPs.
